# Supplementary material for: Identifying the Translatome of Mouse NEBD-Stage Oocytes via SSP-Profiling; A Novel Polysome Fractionation Method
Source: Int J Mol Sci. 2020 Feb 13;21(4):1254. doi: 10.3390/ijms21041254 (PMC7072993; doi:10.3390/ijms21041254)
Supplement: Supplementary file 1 [file ijms-21-01254-s001.zip › ijms-702486 suppl for final/Figures S1-S7_proofs.pdf]

Figure S1:

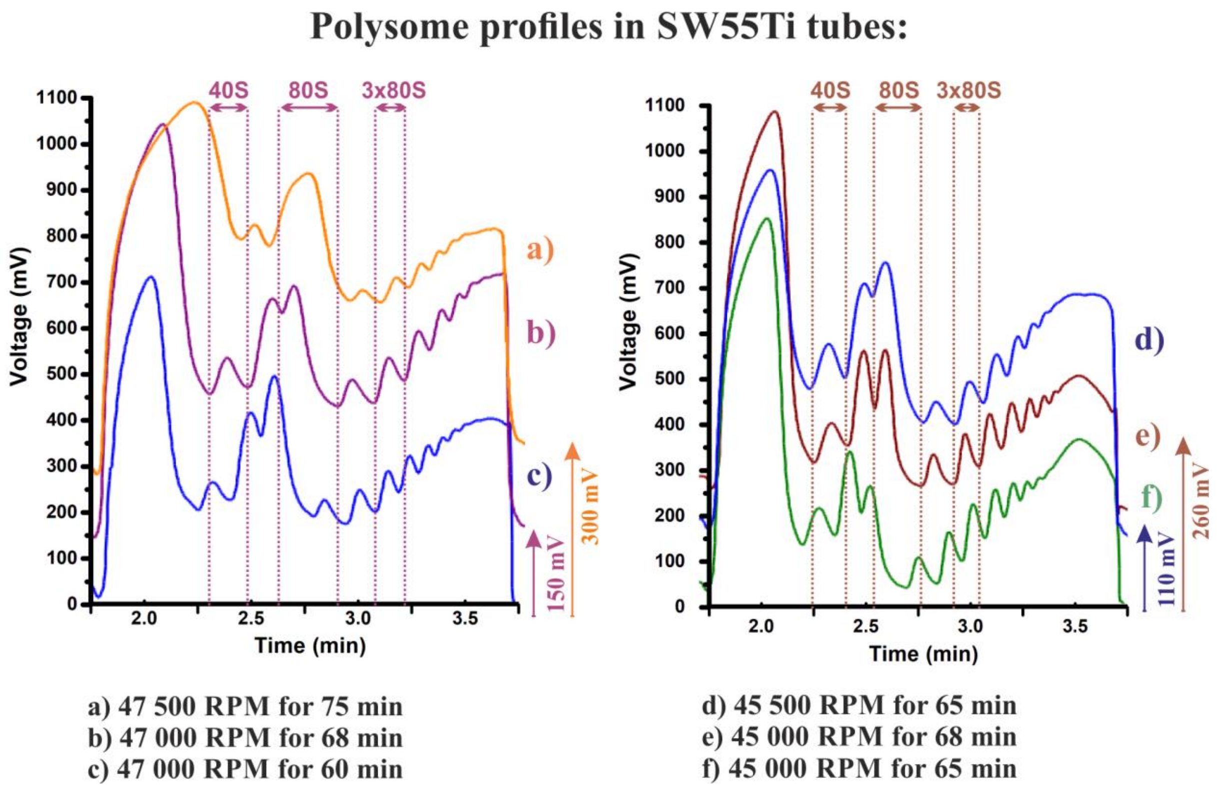

Figure S1: Influence of different centrifugation times and speeds on polysomal profile performance in SW55Ti centrifugation tubes and rotor (a - f). Dotted lines demarcate positions of individual peaks denoted above double-arrowed lines in polysome profile under the letter b) (in violet) and e) (light brown), respectively. 40S, small ribosomal subunit; 80S, monosome; 3 x 80S; three monosomes.

Figure S2:

Figure S2: Spectrophotometric measurement of quantity and purity of isolated RNA obtained from polysome profile fractions of HEK-293 human cell line and yeast strain W303 bearing thermosensitive mutation in guanylyltransferase gene, which was cultivated either at permisive 24°C or restrictive 37°C. Collected fractions were analysed by RNA electrophoresis and the result is provided in Figure 1.

SW55Ti	HEK-293			ceg1-24°C			ceg1-37°C		
Fraction	ng/ul	260/280 nm	260/230 nm	ng/ul	260/280 nm	260/230 nm	ng/ul	260/280 nm	260/230 nm
F1	268,26	1,69	9,04	370,67	1,58	21,93	189,49	1,73	10,42
F2	270,46	1,7	182,21	294,55	1,67	11,83	179,91	1,7	8,08
F3	55,97	1,95	4,94	132,88	1,71	7,4	49,08	1,83	5,57
F4	86,36	1,92	7,26	82,49	1,97	21,83	50,77	1,93	8,63
F5	121,24	1,9	5,06	70,08	2,03	9,11	51,31	1,91	4,49
F6	246,33	1,76	9,48	140,4	1,96	14,2	95,5	1,75	10,46
F7	935,32	1,76	4,66	631,61	1,85	4,67	152,35	1,87	9,38
F8	178,59	1,79	7,05	182,75	1,87	11,32	72,26	1,7	4,25
F9	128,99	1,81	5,7	387,88	1,75	19,7	31,84	1,58	5,19
F10	168	1,81	5,67	536,34	1,87	4,5	26,62	1,56	4,96
F11	179,42	1,79	6,94	384,66	1,81	24,11	44,5	1,45	4,19
F12	245,68	1,78	10,52	306,34	1,85	14,26	21,24	1,58	3,26
F13	450,26	1,75	2,45	317,78	1,88	10,96	23,17	1,34	3,02
F14	493,2	1,72	10,44	393,4	1,85	20,68	17,21	1,54	3,66
F15	532,98	1,74	12,06	183,75	1,86	14,78	45,13	1,25	3,79
F16	580,31	1,68	12,57	81,71	1,8	6,54	13,56	1,38	3,29
F17	380,26	1,68	12,73	37,22	2,21	11,27	8,87	1,2	1,33

SW41	HEK-293			ceg1-24°C			ceg1-37°C		
Fraction	ng/ul	260/280 nm	260/230 nm	ng/ul	260/280 nm	260/230 nm	ng/ul	260/280 nm	260/230 nm
F1	205,83	0,9	0,3	442,14	1,2	41,51	101,03	1,37	5,58
F2	13,9	0,67	84,79	353,2	1,38	21,94	202,75	1,53	10,04
F3	4,85	0,5	0,56	39,85	1,56	1,48	13,07	0,92	3,42
F4	6,48	1,01	10,27	49,24	1,63	1,74	17,84	1	2,47
F5	46,17	1,79	9,46	36,4	1,91	1,54	43,69	1,6	8,93
F6	100,03	1,86	6,12	21,03	2,32	1,07	27,51	1,56	6,64
F7	203,1	1,63	6,39	94,94	1,79	2,56	102,53	1,68	6,71
F8	317,92	1,75	12,84	27,99	1,86	1,23	107,17	1,75	9,73
F9	379,2	1,65	15,44	236,91	1,83	9	71,7	1,63	5,55
F10	202,68	1,79	6,75	339,34	1,8	12,15	-3,56	92,45	1,25
F11	122,99	1,7	6,76	46,02	1,83	1,79	11,51	1,4	7,26
F12	150,98	1,79	7,74	110,09	1,86	3,03	2,89	1,28	1,23
F13	158,32	1,7	5,94	54,11	1,87	2,01	8,12	1,48	2,36
F14	273,75	1,47	4,37	81,36	1,84	2,41	26,74	1,32	7,09
F15	354,72	1,71	12,67	91,12	1,87	3,7	15,03	1,1	7,37
F16	430,29	1,7	13,79	111,5	1,76	3,17	10,37	1,31	2,72
F17	452,44	1,69	15,43	146,11	1,85	4,32	9,33	1,1	7,23
F18	396,12	1,7	12,13	125,29	1,9	3,52	8,38	1,37	6,44
F19	270,22	1,77	7,38	184,89	1,86	4,32	21,94	1,29	7,53
F20				129,06	1,92	3,31	14,83	1,3	22,61
F21				101,88	1,93	3,11	5,83	1,25	1,45
F22				30,96	2,12	1,23			

SW55Ti

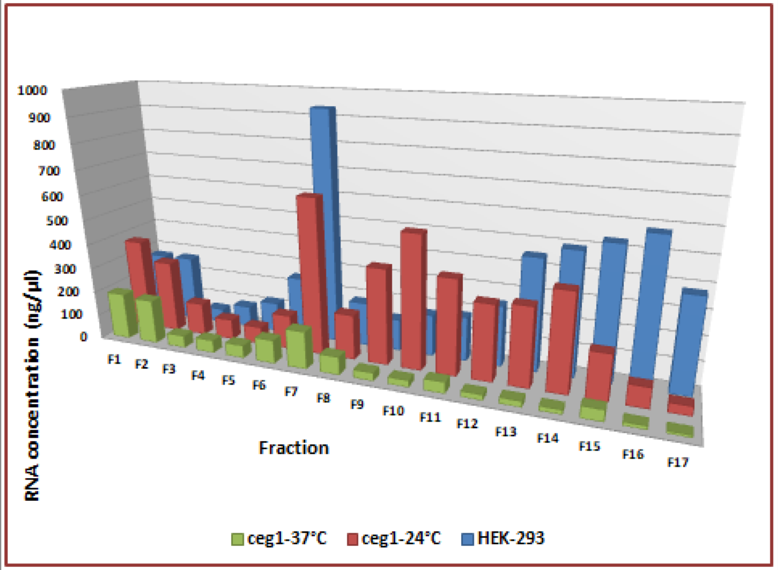

SW41Ti

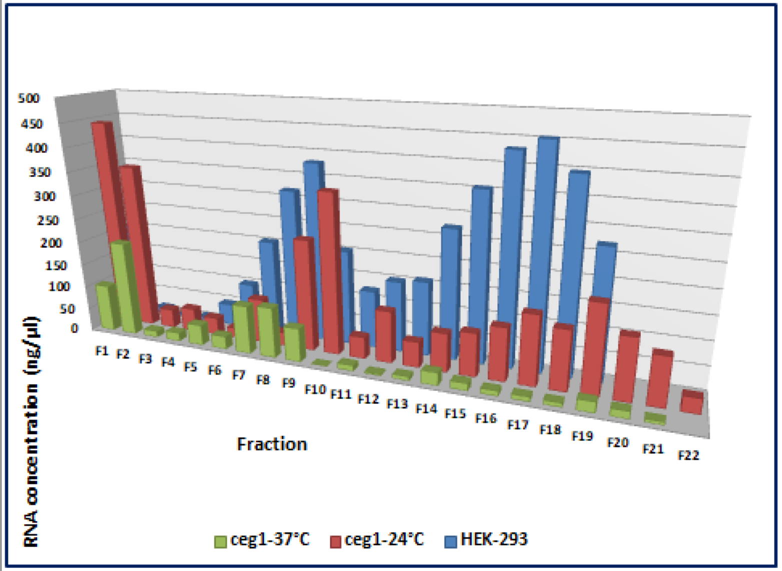

Figure S3:

HEK-293 and NEBD-stage lysates were prepared **(a)** after counting the number of HEK-293 cells prior to lysis (the number of oocytes was fixed at 220). The initial OD_{260nm} of the HEK-293 lysate was measured using a Nanodrop (for NEBD-oocytes this was not technically applicable), before performing HEK-293 lysate serial dilutions in the range from 100x – 10 000x. Additionally, 19 ul of each dilution was subjected to RNA isolation and the RNA concentrations were determined using Agilent BioAnalyzer and compared with RNA content of NEBD-stage oocyte lysate.
We performed qRT-PCR on the HEK-293 polysome (10 000x) fractionation **(b)** and NEBD-stage oocytes **(d)**. In parallel, we prepared reverse transcription controls **(c) and e))** to measure any contaminating DNA in the obtained polysome profile fractions (i.e. the same RNA volume of each fraction was used to prepare reverse transcription reactions with and without enzyme and the individual samples were subsequently co-processed equally).

a)

Cell numbers and lysate volumes									
	dish	confluency	cell number	total lysate volume (ul)	lysate OD (ng/ul, Nanodrop)	volume of lysate loaded on gradient (ul)	Cell number loaded on gradient	volume of lysate used for RNA isolation/serial dilutions (ul)	Cell number in lysate used for RNA isolation (in undiluted)
**HEK-293**	1x15cm	80-90%	2,31E+08	770	1470	190	5,69E+07	19ul	5,69E+06
**NEBD-oocytes**	NA	NA	220	330	NA	300	200	30 ul	20
**Total RNA content determined by Agilent - picochip and rRNA/rDNA copy numbers**									
	dilution factor	total amount (pg in 20 ul)	Corresponding number of cells	18S rRNA copy number in whole	28S rRNA copy number in whole profile	18S rDNA copy number in whole profile	28S rDNA copy number in whole profile	18S rRNA/rDNA ratio	28S rRNA/rDNA ratio
**HEK-293**	100x	1290	5,69E+04						
	1000x	262	5,69E+03						
	10000x	47	5,69E+02	2,11E+08	2,41E+08	6,36E+03	2,85E+03	3,32E+04	8,47E+04
**NEBD-oocytes**	NA	14	2,00E+01	2,79E+07	2,85E+07	4,06E+02	3,69E+02	6,88E+04	7,73E+04

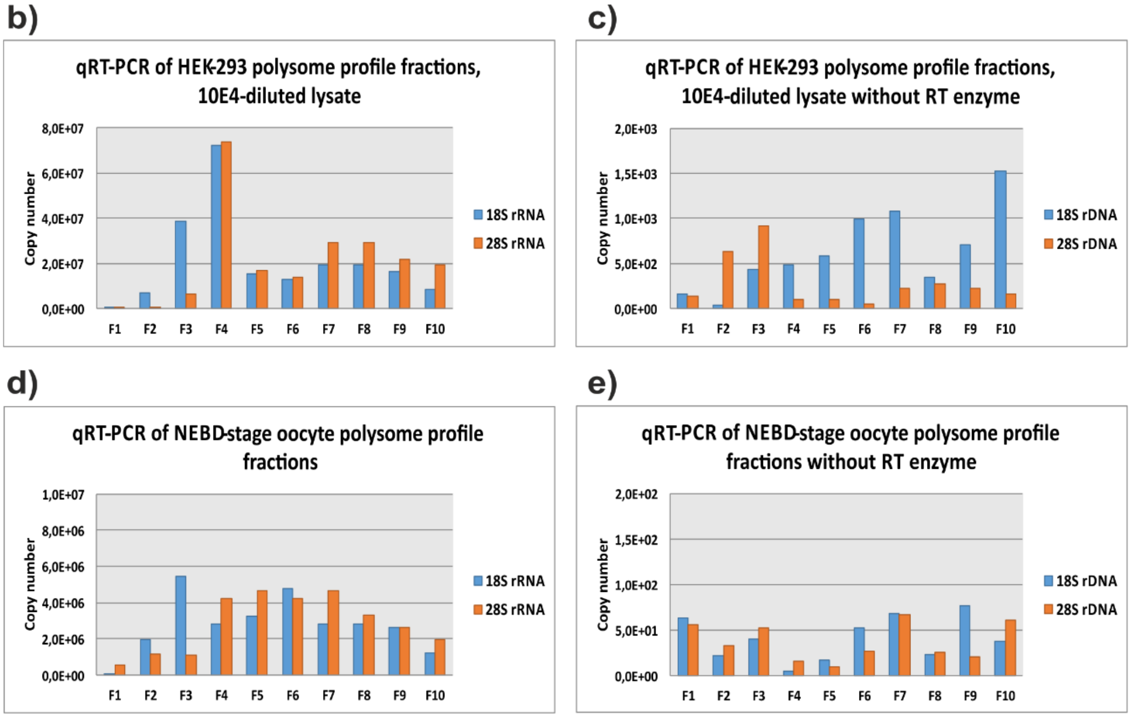

Figure S4:

qRT-PCR-based visualizations of individual replicate/ source polysome profiles used for Figure 2c and Figure 3.

- in figure 2c and 3a:

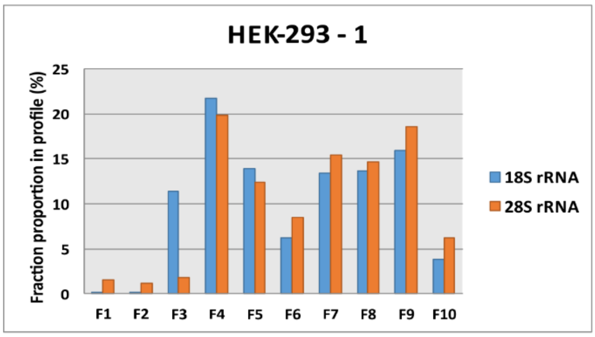

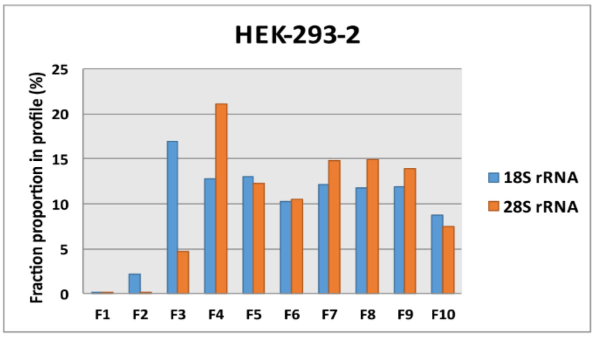

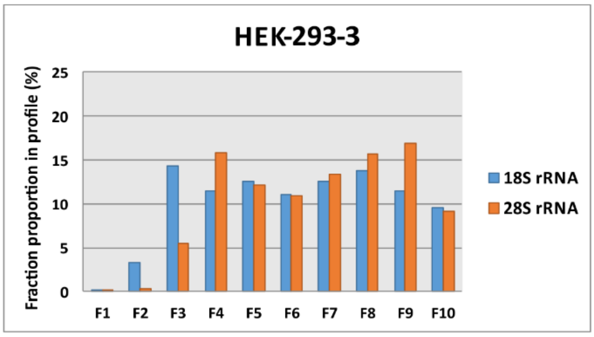

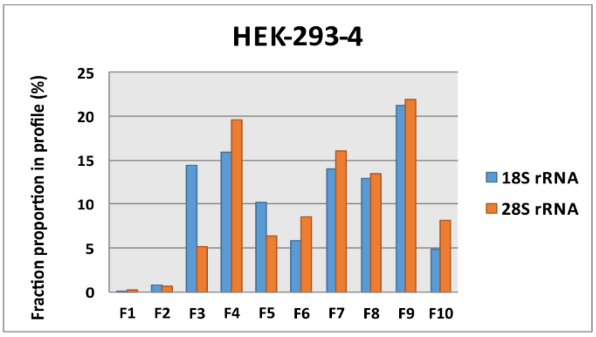

- in figure 2c:

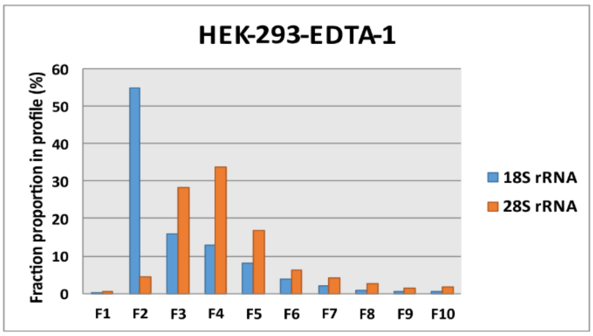

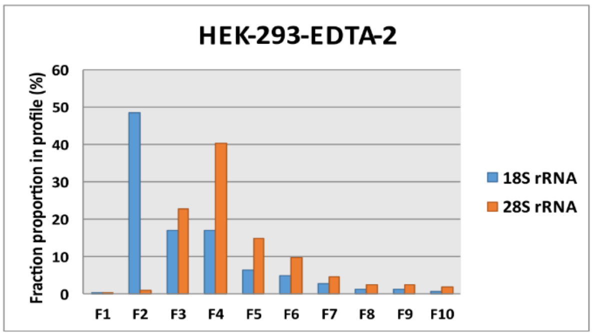

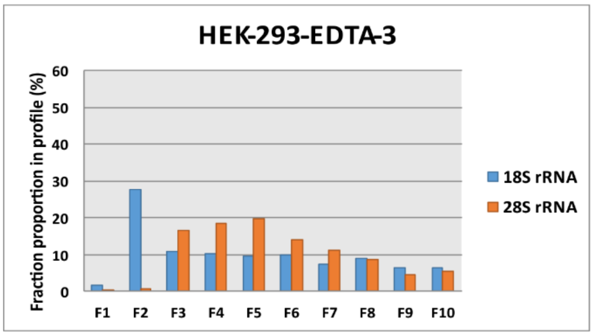

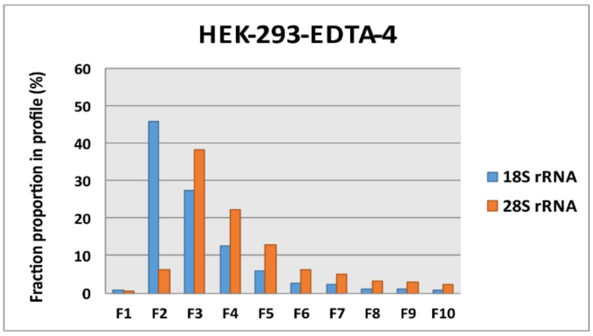

- in figure 2c:

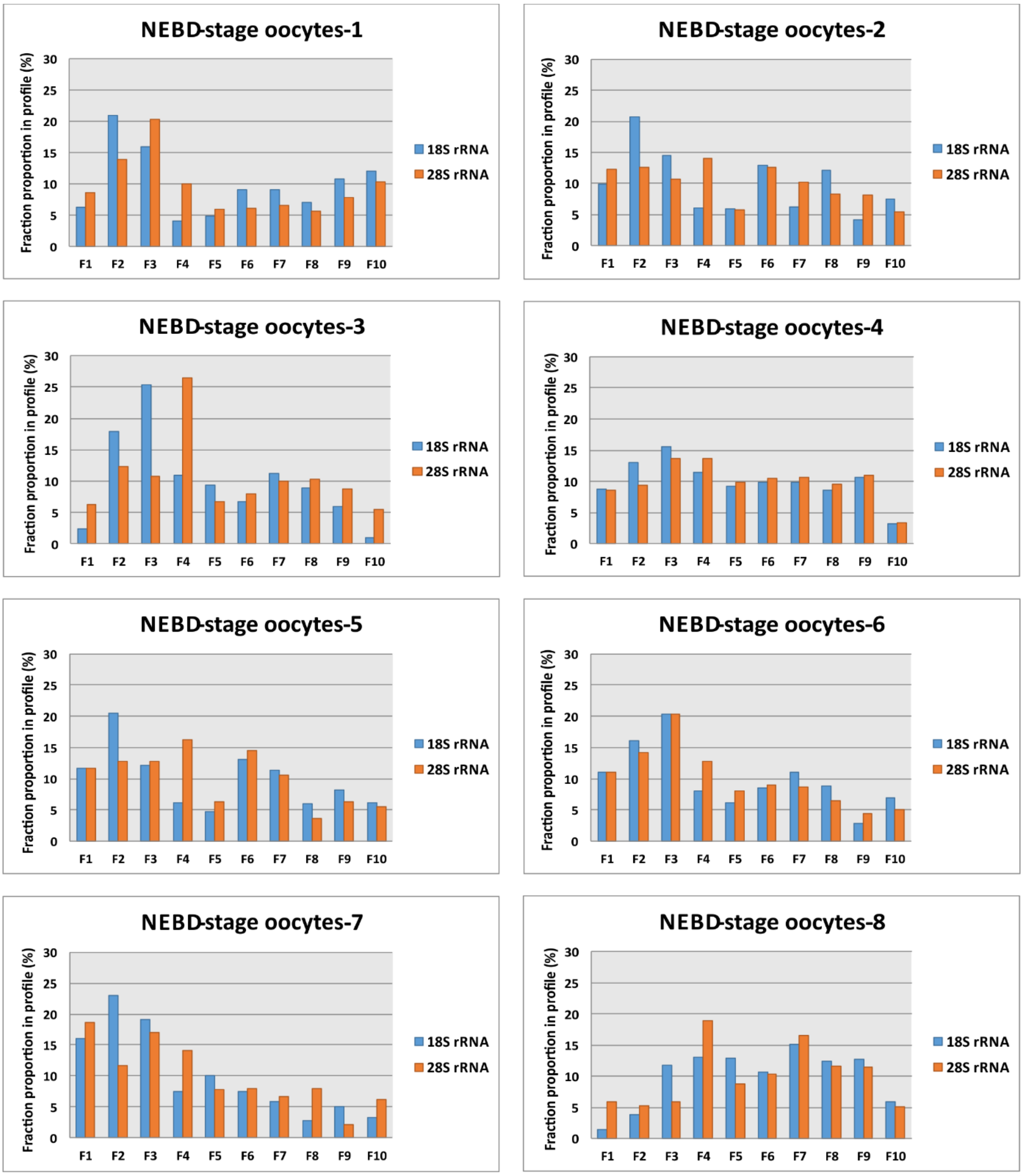
NEBD-stage oocytes-1
NEBD-stage oocytes-2
NEBD-stage oocytes-3
NEBD-stage oocytes-4
NEBD-stage oocytes-5
NEBD-stage oocytes-6
NEBD-stage oocytes-7
NEBD-stage oocytes-8
Fraction proportion in profile (%)
0
5
10
15
20
25
30
F1
F2
F3
F4
F5
F6
F7
F8
F9
F10
18S rRNA
28S rRNA

- in figure 3b:

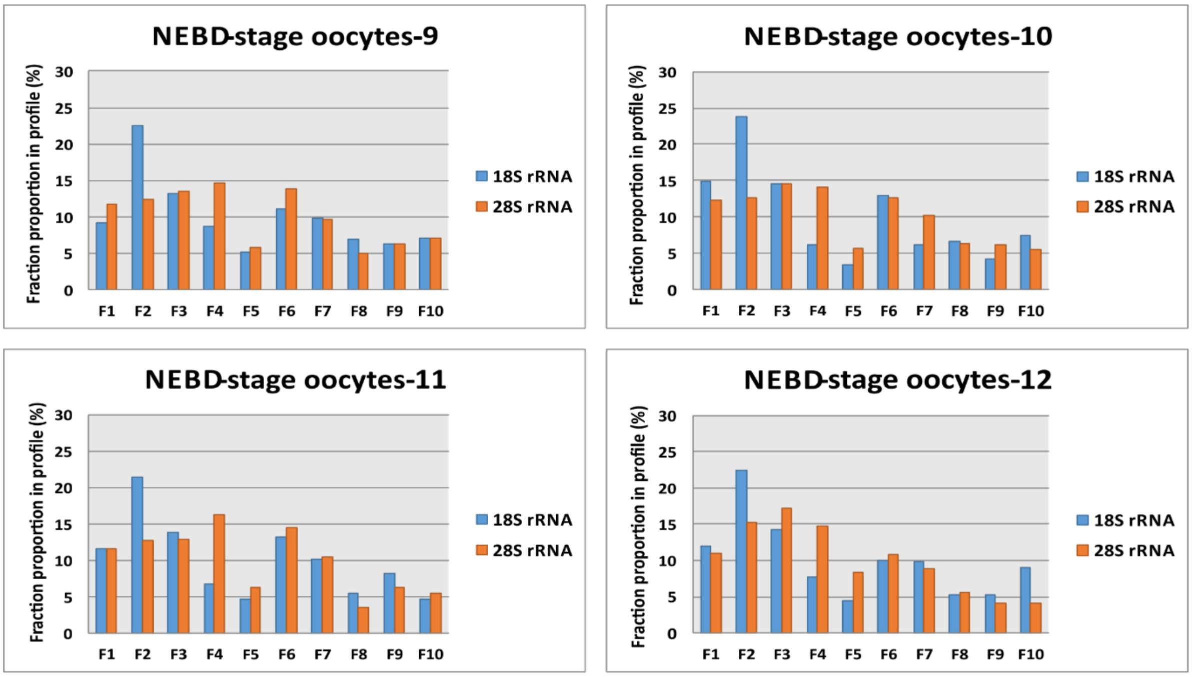
NEBD-stage oocytes-9
NEBD-stage oocytes-10
NEBD-stage oocytes-11
NEBD-stage oocytes-12
Fraction proportion in profile (%)
0
5
10
15
20
25
30
F1
F2
F3
F4
F5
F6
F7
F8
F9
F10
18S rRNA
28S rRNA

- in figure 2c:

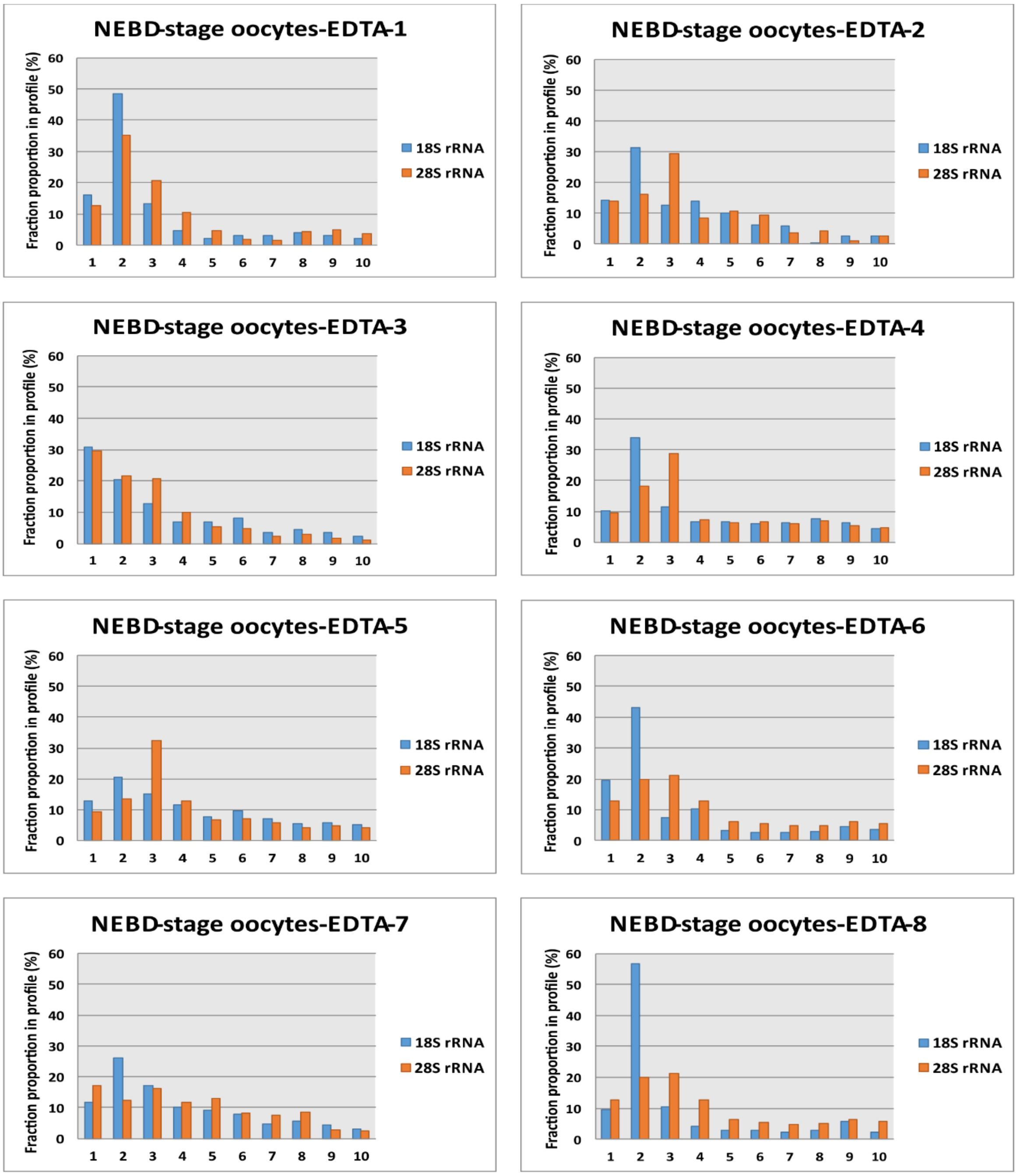
NEBD-stage oocytes-EDTA-1
NEBD-stage oocytes-EDTA-2
NEBD-stage oocytes-EDTA-3
NEBD-stage oocytes-EDTA-4
NEBD-stage oocytes-EDTA-5
NEBD-stage oocytes-EDTA-6
NEBD-stage oocytes-EDTA-7
NEBD-stage oocytes-EDTA-8
Fraction proportion in profile (%)
18S rRNA
28S rRNA
0
10
20
30
40
50
60
1
2
3
4
5
6
7
8
9
10

- in figure 3c:

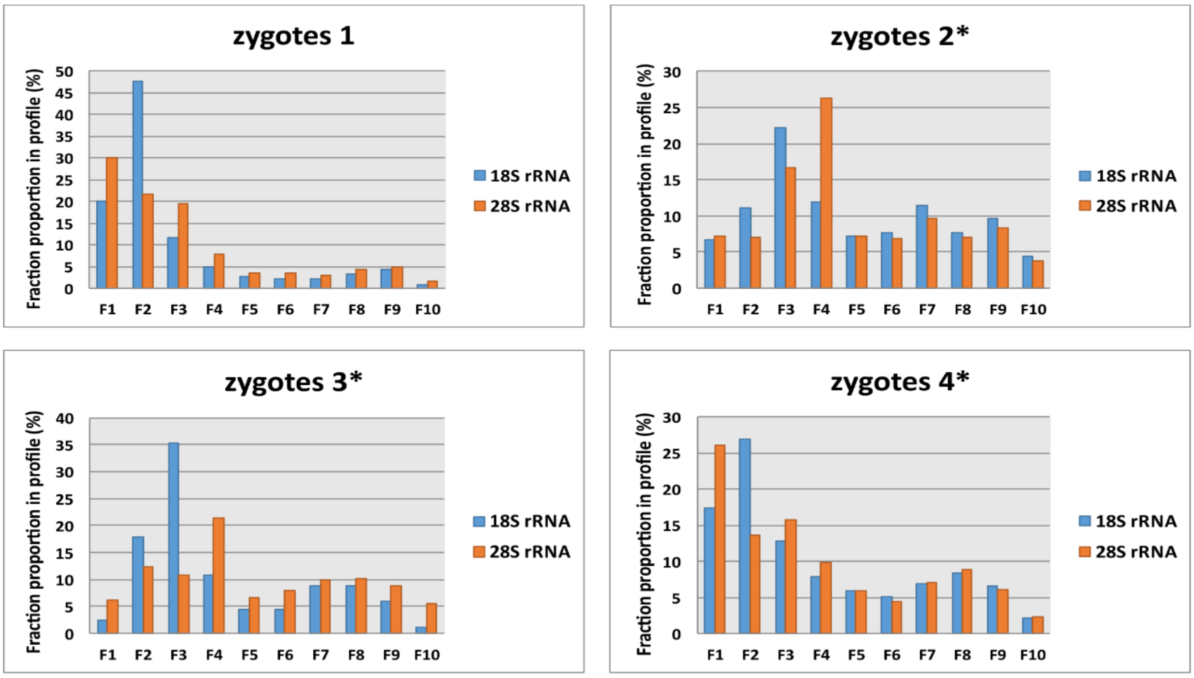

* zygotic samples selected for RNA-seq (performed by Dr. Ch.-J. Lin, see also Figure S5)

- in figure 3d:

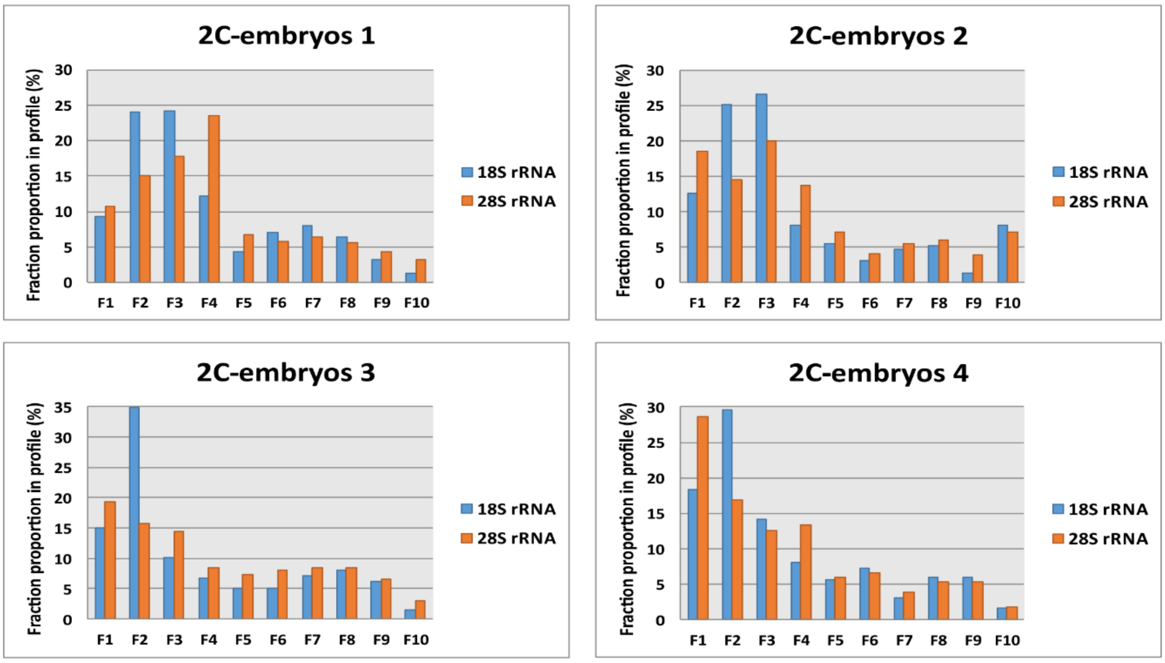

Figure S5:

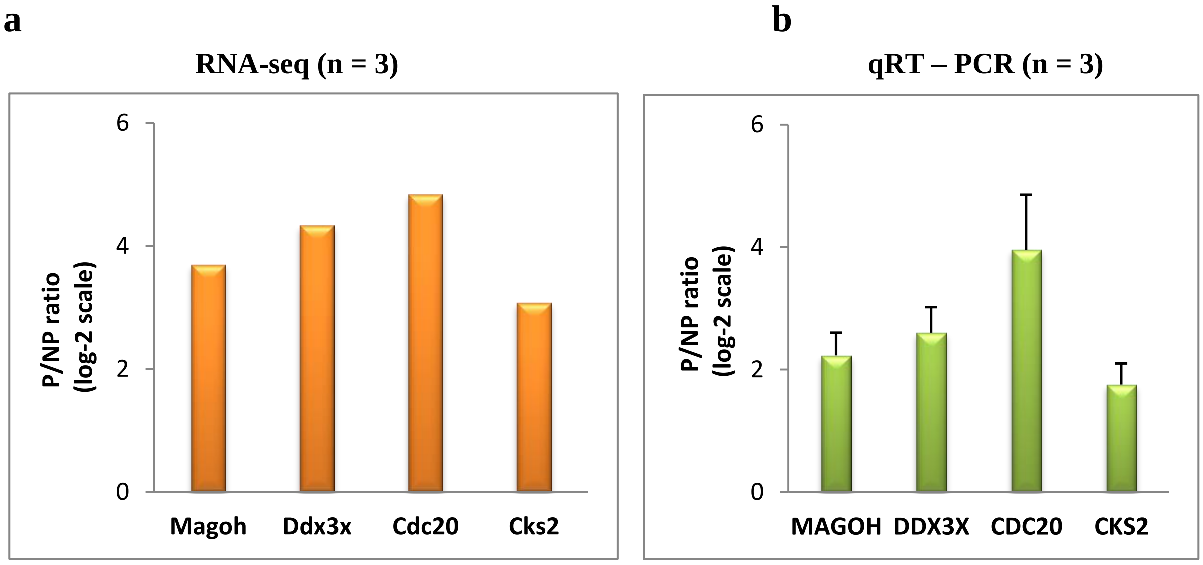

Figure S5: Validation of the selected polysome-enriched zygotic mRNAs.

a) Fold changes of the relative enrichment of polysome-bound mRNAs from RNA-seq datasets. RNA-seq was performed with three biological replicates of samples of 200 zygotes. qRT-PCR visualization of respective polysome profiles is provided in Figure 3B. P/NP ratio was calculated from mean FPKM values in P and NP datasets for particular mRNA.

b) qRT-PCR validation of the selected transcripts. Average values of P/NP ratio are plotted. Copy number of individual transcripts was normalized to mean copy number of reference transcripts *Hprt1* and *H2A.1* before calculating the ratio. Experiments were performed in 3 biological replicates. Sequences of used primers are listed in Supplementary dataset S4. Error bars refer to +SD.

Figure S6:

Plots represent the P/NPs or FPKM values in the P and the NP datasets of individual transcripts according to their lengths.

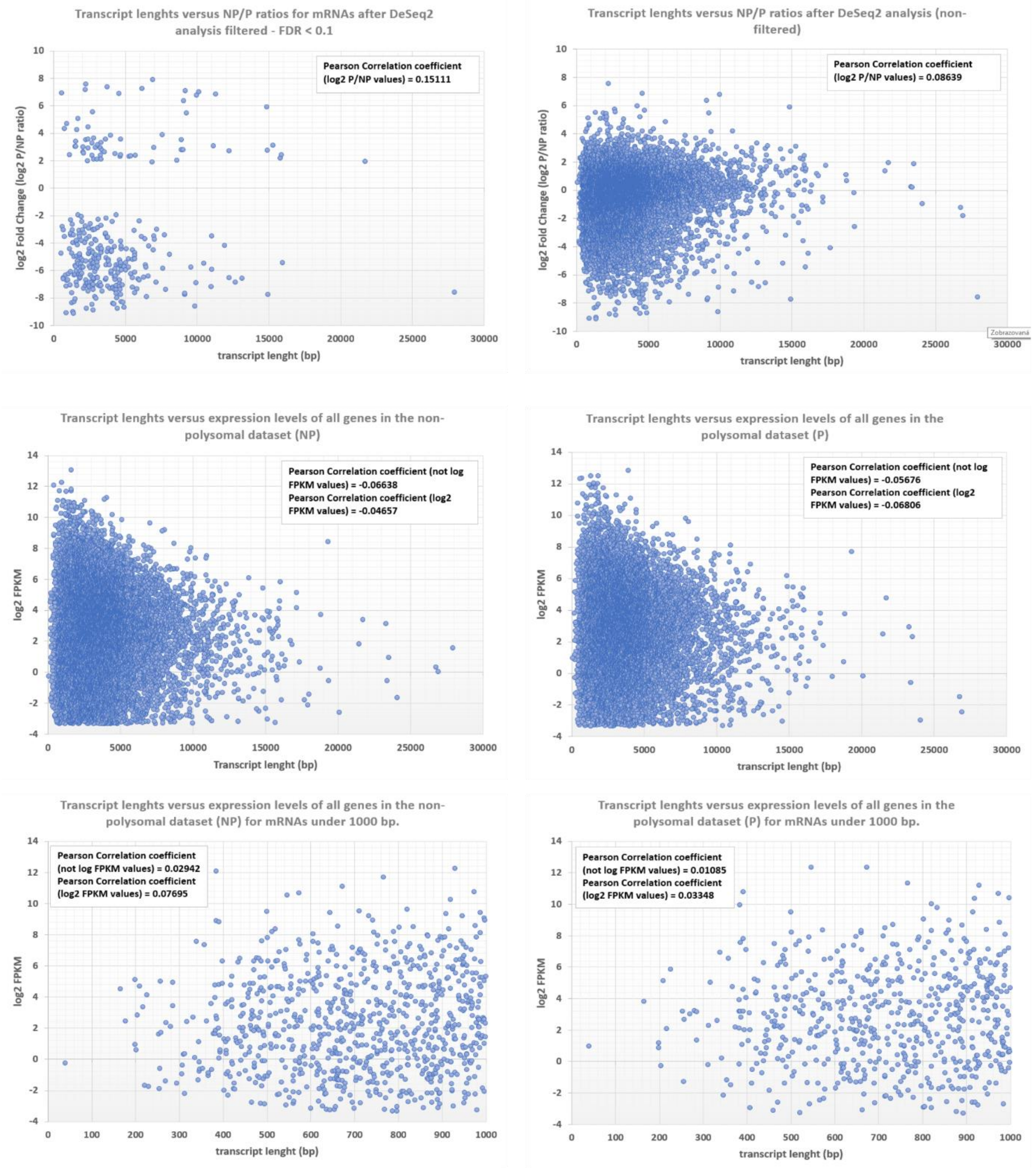

Figure S7:

Reduced-sized-, 5-ml- SW55Ti ultracentrifugation tubes can accommodate enough cell lysate to detect moderately abundant protein in polysome profile fractions.

Aim:

We wanted to confirm that our reduce-sized polysome profiling method is compatible with such western blot analysis and to further test, which concentration of KCl in the sucrose gradient is optimal to detect specific ribosome/polysome interaction with assayed protein. It is known that the gradual increase in salt concentration, first causes the removal of non-specifically-bound proteins, then the disassembly of vacant 80S ribosomes, and eventually to the stripping of specifically interacting protein partners [1]. We therefore chose to assay the association of eukaryotic translational initiation factor 4E (eIF4E), which is the major cap-binding protein and should sediment with its highest detectable signal at the right side of the 40S peak (where the 48S pre-initiation complex sediments) and in lesser extent in the monosomal and polysomal fractions.

Results:

To test this assumption, HEK-293 cell lysate was prepared in a buffer containing 62.5 mM KCl but then loaded on a series of 10-50% sucrose gradients with gradually increasing KCl concentrations, ranging from 62.5 mM to 200 mM (Figure herein). In this case, ultra-centrifugation was performed for 65 minutes at 47,500 RPM at 4°C as was evident from the lack of very high-molecular polysomes in the profiles (compare Fig.1 and Figure herein and see Supplementary Figure S1). eIF4E was found to be most abundant in fractions 3 – 5 and 2 - 4 in the gradients containing 62.5 mM KCl and 100 mM KCl, respectively, with the fraction 4 most likely containing the majority of 48S complexes. The inclusion of 140 mM and 200 mM KCL into the sucrose gradients resulted in detection of the eIF4E only in the fractions 1 and 2, which corresponded to the position of the loaded sample and did not contain any ribosomal complexes (see Figure herein). An overlay of polysomal profiles obtained in gradients of varying KCl demonstrates the effect of salt increasing concentration on the disassembly of vacant 80S ribosomes, as is evident from gradually increasing peaks corresponding to the 40S and the 60S ribosomal subunits.

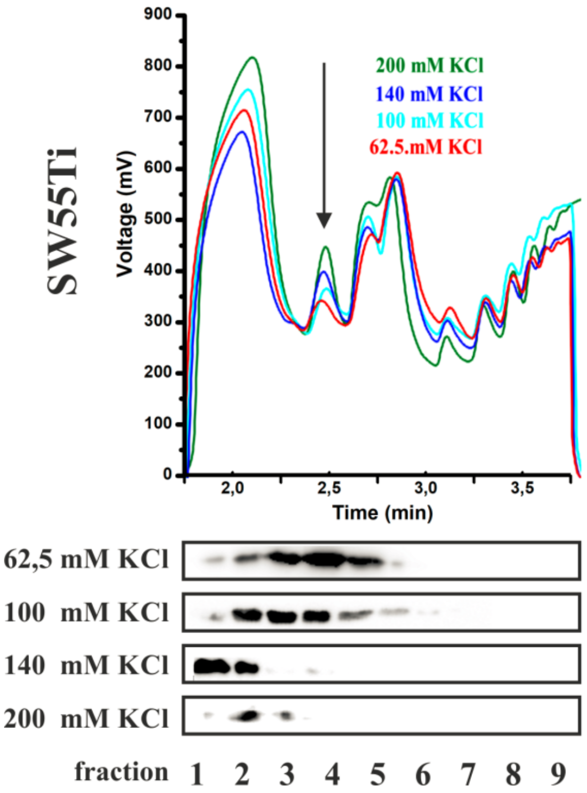

Suitability of SW55Ti-based setup for Western blot analysis. HEK-293 lysate was loaded onto 10 – 50% sucrose gradients with gradually increasing concentration of KCl (62,5 – 200 mM; as indicated). Polysome profiles obtained (upper traces) and western blot data probing the eukaryotic translation initiation factor 4E (eIF4E) in fractionated profiles (lower panel) are presented.

Conclusion and discussion:

The intensity of the eIF4E western blot signal indicates that the reduced volume SW51Ti tube specific protocol can accommodate enough cell lysate to detect any moderately abundant protein.

We also demonstrated that the KCl concentration used, ultimately affects the western blot result. The effect of salt concentration has been previously reported [1] but it is often underappreciated and we suggest that this kind of control (*i.e.* employing various salt concentrations) is advisable to be sure that the studied protein is specifically bound to ribosomes/polysomes.

Methods:

Proteins from polysome profile fractions were purified according to [2] by TCA – isopropanol procedure and dissolved in 1x Laemmli buffer supplemented with 50 mM TCEP (Sigma-Aldrich) and 1x Complete protease inhibitor cocktail (Sigma Aldrich). Western blot analysis was performed as previously described in [3] with the following modifications: proteins were transferred on PVDF membrane (Biorad); chemiluminiscence signal was acquired on ImageQuant LAS4000 Series imaging system (GE Healthcare Life Sciences); primary antibody was rabbit polyclonal eIF4E1 (E5906) from Sigma Aldrich and secondary antibody was goat anti-rabbit IgG-HRP (sc-2004) from Santa Cruz.

References:

1. Sivan, G.; Kedersha, N.; Elroy-Stein, O. Ribosomal slowdown mediates translational arrest during cellular division. *Molecular and cellular biology* **2007**, *27*, 6639-6646, doi:10.1128/mcb.00798-07.
2. Frydryskova, K.; Masek, T.; Borcin, K.; Mrvova, S.; Venturi, V.; Pospisek, M. Distinct recruitment of human eIF4E isoforms to processing bodies and stress granules. *BMC molecular biology* **2016**, *17*, 21, doi:10.1186/s12867-016-0072-x.
3. Zamostna, B.; Novak, J.; Vopalensky, V.; Masek, T.; Burysek, L.; Pospisek, M. N-terminal domain of nuclear IL-1alpha shows structural similarity to the C-terminal domain of Snf1 and binds to the HAT/core module of the SAGA complex. *PloS one* **2012**, *7*, e41801, doi:10.1371/journal.pone.0041801.
